# Dyslipidemia in Myasthenia Gravis: A Systematic Review and Meta-Analysis

**DOI:** 10.3390/medicina61061067

**Published:** 2025-06-10

**Authors:** Vasileios Giannopapas, Vassiliki Smyrni, Georgia Papagiannopoulou, Stavroula Salakou, Dimitrios K. Kitsos, Ilianna Bethani, Christina Zompola, John S. Tzartos, Georgios Tsivgoulis, Sotirios Giannopoulos, Maria Kosmidou

**Affiliations:** 1 Second Department of Neurology, Attikon University Hospital, School of Medicine, National and Kapodistrian University of Athens, 12462 Athens, Greece; bgiannopapas@gmail.com (V.G.); b.smyrni@hotmail.com (V.S.); georgiapap22@hotmail.com (G.P.); ssalakou@gmail.com (S.S.); dkitsos@icloud.com (D.K.K.); ilianna.bethani@gmail.com (I.B.); chriszompola@yahoo.gr (C.Z.); jtzartos@gmail.com (J.S.T.); tsivgoulisgiorg@yahoo.gr (G.T.); 2 Third Department of Internal Medicine, Aristotle University of Thessaloniki, 54124 Thessaloniki, Greece; mskosmidou@gmail.com

**Keywords:** myasthenia gravis, dyslipidemia, autoimmune disorders, prevalence

## Abstract

*Background and Objectives*: Myasthenia Gravis (MG) comprises an autoimmune disorder marked by muscle weakness and fatigue. MG has been associated with comorbid conditions, including dyslipidemia (DL), which may exacerbate the clinical burden of MG and impact the overall quality of life. *Materials and Methods*: This systematic review and meta-analysis aimed to assess the prevalence of DL in patients with MG. Following PRISMA guidelines, a comprehensive search was conducted in the MEDLINE, Scopus, and Google Scholar databases. Primary outcomes included the pooled prevalence of DL in MG patients, and the relative risk of DL compared to healthy controls. *Results*: Nineteen studies involving 98,947 MG patients were analyzed. The pooled prevalence of DL was 23.64% (95% CI: 17.01–30.98). The relative risk of DL in MG patients versus controls was 1.13 (95% CI: 0.53–2.41), indicating no significant increase. Meta-regression revealed a positive correlation between DL prevalence and MG onset age (β = 0.02, *p* < 0.001), with a 2% rise in DL prevalence per 1-year increase in onset age. Regional subgroup analysis showed a statistical trend of higher DL prevalence in the USA (33.02%) compared to Asia (19.89%) and Europe (17.5%). *Conclusions*: This study found that approximately one in four MG patients has comorbid DL, with MG onset age significantly influencing DL prevalence. These findings highlight the need for personalized management strategies and evaluations (e.g., statins, LP(a) levels). Further research is warranted to elucidate the pathophysiological links between MG and DL.

## 1. Introduction

Myasthenia Gravis posits a rare, chronic, neuromuscular disease affecting approximately 12.4 per 100,000 persons globally [1]. MG is characterized by the production of pathogenic autoantibodies that target key components of the neuromuscular junction, leading to fluctuating weakness in striated muscles. This weakness predominantly affects the extraocular, bulbar, limb, and axial muscles, resulting in symptoms such as ptosis, diplopia, dysphagia, and generalized fatigue [1,2].

Despite the fact that the exact underlying pathomechanisms of MG remain incompletely comprehended, current evidence suggests that autoantibodies play a central role in disrupting postsynaptic function. More specifically, pathogenic autoantibodies disrupt postsynaptic transmission, leading to the internalization of anti-acetylcholine receptors (AChRs), direct blockade of AChR function, or interference with the agrin/LRP4/MuSK/Dok7/rapsyn signaling cascade [3]. These mechanisms compromise neuromuscular junction signaling, resulting in clinical manifestations such as variable muscle weakness and increased fatigability [3].

In addition to its autoimmune etiology, MG has been increasingly associated with comorbid conditions, including dyslipidemia (DL). DL is a condition caused by abnormal lipid levels in the bloodstream, serving as a crucial risk factor of cardiovascular diseases, such as diabetes [4,5,6]. Several studies suggest a potential association between DL and autoimmune disease, such as MG; however, the exact nature of this relationship remains incompletely comprehended [7,8].

Although a rare disease, the incidence of MG has more than doubled in the last 20 years, raising the need for enhanced diagnostic approaches, improved therapeutic interventions, and a deeper understanding of the environmental, genetic, and immunological factors driving this increase [9]. To date, the exact prevalence of DL in the MG population remains inconclusive, underlying a critical gap in the literature that highlights the need for further investigation. Hence, given the growing evidence linking DL to MG, the objective of this systematic review and meta-analysis is to estimate the prevalence of DL in patients with MG, which may provide critical insights into its potential role in disease progression, comorbidities, and overall clinical management.

## 2. Materials and Methods

### 2.1. Standard Protocol Approval-Registrations

The pre-specified protocol of this systematic review and meta-analysis is registered in the Open Search Foundation (available at doi:10.17605/OSF.IO/QWCUY). The results of the present systematic review and meta-analysis are in accordance with the Preferred Reporting Items for Systematic Reviews and Meta Analysis (PRISMA) [10] and were written based on the Meta-Analysis of Observational Studies in Epidemiology proposal (MOOSE). Due to the nature of this study (review), no ethics board approval or written informed consent was required.

### 2.2. Data Sources, Search, and Study Selection

Two major medical databases, MEDLINE PubMed and Scopus, were used in the systematic literature search in addition to the first 200 results of Google Scholar. Two independent reviewers (VG,SG) independently searched the databases using the following terms: “Myasthenia Gravis”, “Dyslipidemia”, “Hyperlipidemia”, “Metabolic syndrome”, “Cardiovascular”. No date or language filtering was applied. The complete search algorithm is provided in the Supplementary Materials. The search spanned from inception (10 December 2024) to 8 January 2025, with no expectation of a repeated search. Case–control studies and observational studies were included. The pre-specified inclusion criteria were (a) adult participants, (b) diagnosis of MG, and (c) reporting of cases of DL or proportion of participants receiving statins.

Studies were excluded if patients (a) were <18 years old, (b) had a concomitant neurological condition, (c) reported cross-sectional data on cholesterol or triglyceride levels, and (d) sample size was less than 30 participants. Additionally, we excluded clinical trials and studies employing purposive sampling.

All retrieved studies were independently assessed by two reviewers (VG,SG) and any disagreements were resolved by the senior author (MK).

### 2.3. Quality Control, Bias Assessment, Data Extraction

The risk of bias for each study was assessed using the Risk of Bias in Non-Randomized studies of Exposure (ROBINS-E) [11]. Quality control and bias assessments were performed by two independent reviewers (VG,SG) and any disagreements were resolved by the senior author (MK).

Data extraction was also performed by the same two independent authors and included study title, author, year of publication, region, sample size, demographic characteristics (age, gender, etc.), disease specific characteristics (type of MG, age of onset, MGFA classification, etc.), proportion of participants with DL, and proportion of participants receiving statins.

### 2.4. Outcomes

The predefined primary outcome was the proportion of dyslipidemia in patients with Myasthenia Gravis. Secondary outcomes were mean levels of triglycerides, lactate dehydrogenase and low-density lipoprotein in patients with MG, and potential associations between demographic factors (age, gender) and disease specific factors (disease duration, MGFA class, MG-ADL, age of onset) and proportion of DL.

### 2.5. Statistical Analysis

For the aggregated meta-analysis of proportion, the meta-prop function of R-Meta was employed. The random effects meta-analytical model (DerSimonian and Laird) was used to calculate the pooled estimates and the corresponding 95% confidence intervals (95% CI) [12]. Heterogeneity was assessed by I2 values (>50% values >75% were considered to represent substantial and considerable heterogeneity, respectively) [13]. The statistical significance level for the Q statistic was set at 0.1. Publication bias across individual studies was assessed for the primary outcome of interest, using funnel plot inspection as well as the Egger’s linear regression test and the equivalent z test for each pooled estimate, with a two-tailed *p* value < 0.05 being considered statistically significant [14]. All statistical analyses and figure production were carried out using RStudio for IOS v.4.2.3 [R studio/R Meta package] [15].

### 2.6. Data Availability Statement

All data generated or analyzed for this study are included in this article and its Supplementary Materials. Raw data are available in plain text format upon reasonable request from the corresponding author.

## 3. Results

### 3.1. Literature Search

**Figure 1:**
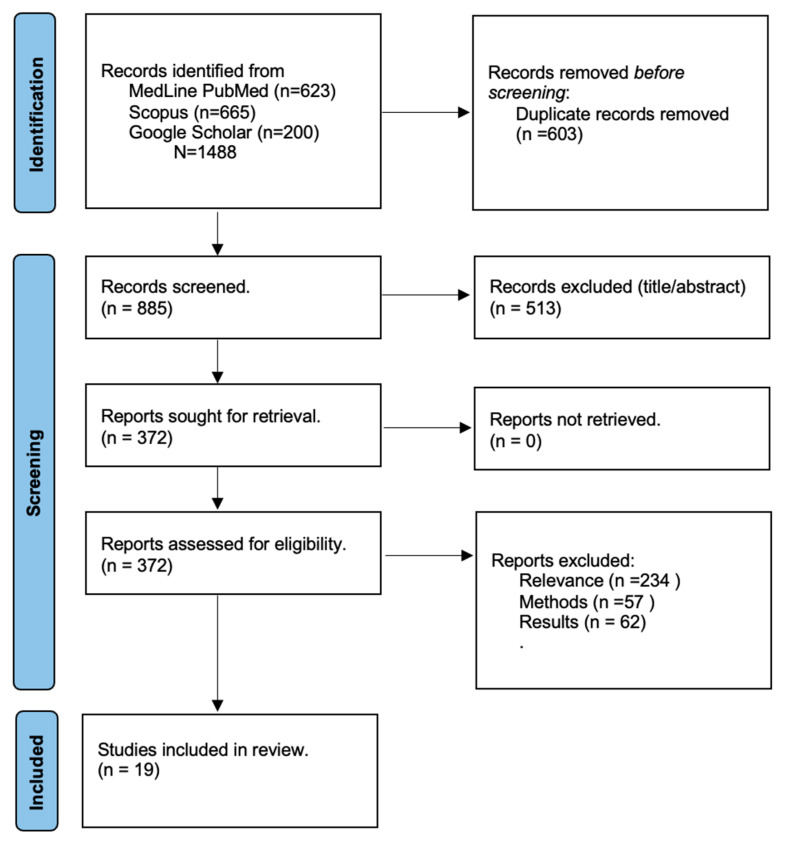
PRISMA flowchart.

A total of 1488 results were retrieved from the systematic literature search. After the removal of duplicate records and the exclusion of irrelevant results based on the title and abstract, 372 results were further assessed. Finally, after the implementation of the pre-specified inclusion–exclusion criteria, 19 studies were included in the synthesis. A graphic presentation of the study selection process is provided in Figure 1.

### 3.2. Risk of Bias Assessment

Risk of bias was assessed using the Robins-E. The included studies that had low-to-moderate risk of bias, mainly due to missing data (Figure S1).

### 3.3. Qualitative Results

A total of 19 studies [16,17,18,19,20,21,22,23,24,25,26,27,28,29,30,31,32,33,34] were included in this systematic review and meta-analysis. The majority of the studies reported data on dyslipidemia in patients with MG as a sample characteristic without specifying DL’s characteristics (type, onset, management type, etc.). In three studies [16,17,20], DL was among the predefined primary outcomes. Oh and colleagues (2008), examined the effects of statins in patients with MG and the risk of statin-induced myopathy and reported worsening MG symptoms, which predominantly involved oculobulbar symptoms, 1 to 16 weeks after statin treatment initiation [16]. Haggard and colleagues (2012) examined the effect of β-glucans on the reduction in Low Density Lipoprotein (LDL) in patients with MG. They observed that in patients with MG, a daily dose of 3 g of β-glucans significantly reduced total cholesterol, LDL-C, and ApoB after 8 weeks without the muscle related side-effects and MG that are observed in patients under statins treatment [17]. Finally, Li and colleagues (2018) investigated the glucose and lipid metabolic disorders in PwMG without glucocorticoid therapy and their relationships with insulin, insulin resistance, muscle strength, serum levels of osteocalcin, and 25-hydroxy-vitamin D in a sample of 102 patients, and observed a high rate of glucose and lipid metabolic. Given the absence of glucocorticoid therapy, the authors hypothesize that the underlying mechanism may be related to insulin resistance secondary to muscle weakness [20].

### 3.4. Quantitative Results

A total of 19 studies [16,17,18,19,20,21,22,23,24,25,26,27,28,29,30,31,32,33,34] comprising 98947PwMG reported data on DL (Table 1). Regarding demographic and MG characteristics, the mean age among included studies ranged from 40 to 64.7 years, while the MG onset age ranged from 44.7 to 70.4 years. In studies that reported MG duration, disease duration ranged from 2.8 to 34.1 years.

**Figure 2:**
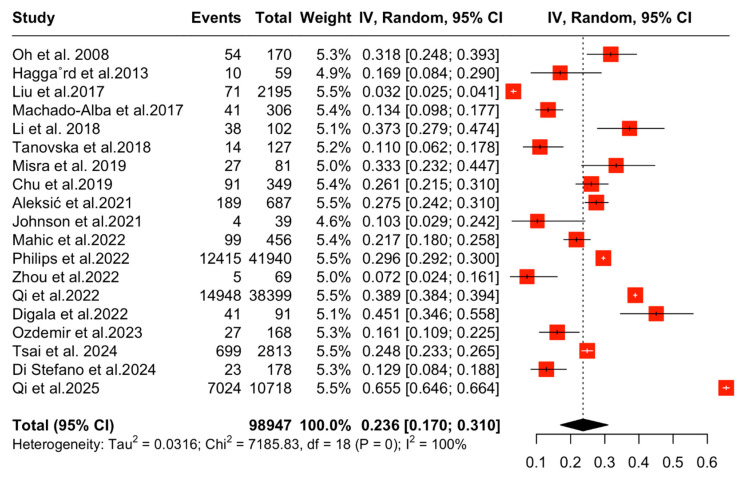
Forest plot: Pooled proportion of DL in PwMG [16,17,18,19,20,21,22,23,24,25,26,27,28,29,30,31,32,33,34].

In regard to the primary outcome of this study, the aggregated pooled prevalence of DL in PwMG was 23.64% (95% CI [17.01,30.98], I^2^ = 99.7%, *p* = 0) (Figure 2).

A subsequent analysis of two studies that reported data regarding DL cases on both PwMG and healthy controls was performed. The aggregated relative risk of DL in PwMG was 1.13 (95%CI [0.53,2.41], I^2^ = 91.6, *p* < 0.001), which indicates that based on the limited available data, PwMG do not have a greater risk of DL compared to the general population.

Potential correlations between the prevalence of DL and demographic characteristics were assessed using meta-regression techniques. In a pool of 6 studies that reported data regarding MG onset age, a statistically significant relationship between onset age and DL proportion was found (β = 0.02, *p* < 0.001), which translates to a 2% increase in DL proportion with every 1-year increase in MG onset age. On the other hand, DL proportion had no statistically significant relationship with either participants’ age at index (*p* = 0.36) or duration of MG (*p* = 0.62).

**Figure 3:**
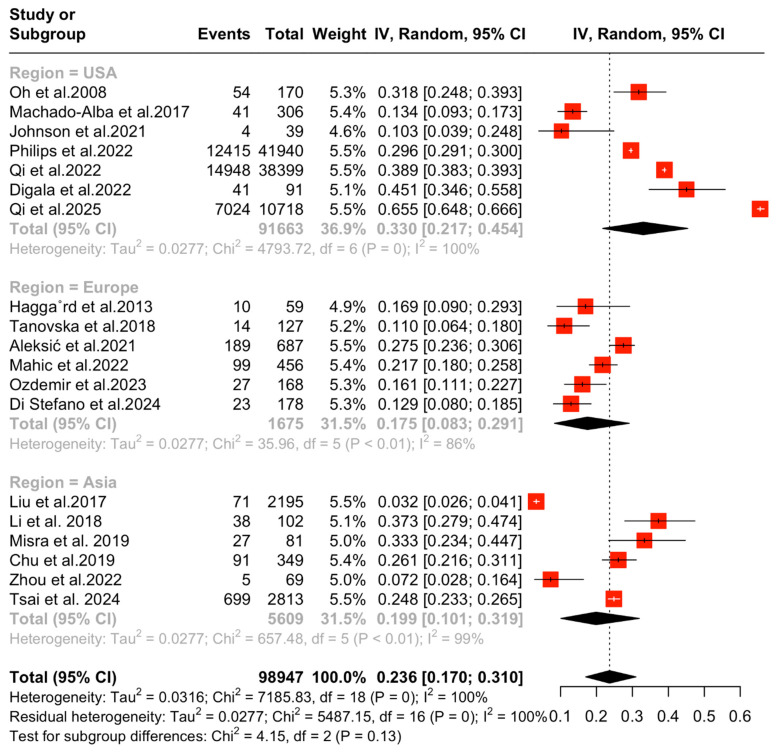
Forest plot: Subgroup differences based on region [16,17,18,19,20,21,22,23,24,25,26,27,28,29,30,31,32,33,34].

Finally, a subgroup analysis based on region (USA, Asia, Europe) revealed a DL proportion of 33.02% (95% CI [21.7, 45.35], I^2^ = 99.9%, *p* = 0), 19.89% (95% CI [10.1, 31.9], I^2^ = 99.2%, *p* < 0.01), and 17.5% (95% CI [21.7, 45.35], I^2^ = 86.1%, *p* ≤ 0.01) for each respective region, with evidence of a statistical trend between groups (*p* = 0.12) that did not pass the statistical significance cut-off point (Figure 3).

### 3.5. Publication Bias

Publication bias was assessed by funnel plot inspection and Egger’s linear regression test, with the included studies presenting with low evidence of publication bias (β = −4.89, *p* = 0.38) (signs of asymmetry, Figure S2).

## 4. Discussion

This systematic review and meta-analysis included a cohort of 98,947 individuals with MG, with a mean age of 53.8 years. Approximately one in four patients with MG had a confirmed diagnosis of dyslipidemia (DL), with the pooled prevalence of DL estimated at 23.64% (17–31%). In the general population, DL rates range from 9% to 53.7%, whereas in the MG population, the DL rates appear comparatively lower. This variability in DL rates has been attributed to two main factors. First, the diagnostic criteria used to define DL play a significant role in determining its reported prevalence. Second, geographical location or region is a critical determinant, as data from the WHO indicate that countries in Asia and the Pacific report lower rates of DL compared to Europe and the United States (WHO, 2016) [35].

Our analysis showed that studies conducted in the US reported the highest prevalence of DL among PwMG (33%), followed by Asia (20%) and Europe (18%). Moreover, the reported data suggest a 2% increase in DL prevalence with each additional year of age at MG onset, which may be attributed to secondary factors associated with DL, such as dietary habits and sedentary lifestyle [36]. It is worth noting that studies evaluating the prevalence of DL in the general population, as well as the majority of studies included in this meta-analysis, lack comprehensive data regarding the etiology and subtype of DL (familial vs. secondary). This raises an important and underexplored question concerning the causal relationship between MG and DL—specifically, whether DL in PwMG is primarily familial, acquired due to comorbidities or lifestyle, or directly disease-related. Despite the growing literature on the immunometabolic interface, there remains a paucity of mechanistic data elucidating the pathophysiological interplay between lipid metabolism and autoimmunity in MG.

Previous studies have identified several factors linking MG and DL, although the directionality and underlying mechanisms remain poorly understood. One of the most consistently reported associations involves genetic predisposition, particularly variations in the human leukocyte antigen (HLA) system and the apolipoprotein E (APOE) gene. Specifically, the HLA-B8-DR3 and HLA-B7-DR2 haplotypes have been associated with earlier disease onset, more severe symptomatology, and a greater likelihood of generalized MG phenotypes [37,38]. These HLA alleles are also linked to immune dysregulation and may contribute to the breakdown of self-tolerance, a critical step in the pathogenesis of autoimmune diseases. In parallel, apolipoprotein E (ApoE) serves as a key regulator of lipid transport and metabolism but also exerts immunomodulatory effects, particularly in the context of T-cell activation and antigen presentation. Among the three major isoforms of ApoE (ε2, ε3, and ε4), the APOE4 allele has been most strongly associated with dyslipidemia due to its role in elevating plasma LDL cholesterol and impairing receptor-mediated lipoprotein clearance. Notably, recent studies have identified APOE4 as the most common isoform among patients with MG, raising the possibility of a shared genetic susceptibility to both lipid dysregulation and autoimmune neuromuscular dysfunction. Furthermore, the presence of APOE4 has been correlated with more aggressive MG phenotypes and poorer response to standard immunosuppressive therapies. These observations suggest a potential genotype–phenotype correlation that merits further exploration in both mechanistic and longitudinal clinical studies [38].

Second, several studies have indicated that individuals with cardiovascular risk factors, such as hypertension and hypercholesterolemia, have an increased likelihood of being diagnosed with MG. This association may be potentially attributed to the use of beta-blockers, which have been linked to clinical worsening and an elevated risk of myasthenic crisis. Additionally, chronic vascular inflammation and endothelial dysfunction in patients with cardiovascular comorbidities could contribute to peripheral immune dysregulation, potentially lowering the threshold for autoimmunity [32,39,40]. In a seven-year retrospective study, Zhang et al. (2024) [41] demonstrated that lipoprotein(a) [LP(a)] levels were correlated with an increased risk of clinical worsening and myasthenic crisis. LP(a) levels within the range of 7–58 nmol/L were significantly associated with a higher one-year risk of MG exacerbation. This association may be explained by several mechanisms. LP(a) has been implicated in pro-inflammatory signaling via the accumulation of oxidized phospholipids, contributing to T- and B-cell activation [41]. Additionally, LP(a) may compromise vascular function through endothelial nitric oxide synthase (eNOS) inhibition and interfere with lipid raft integrity in immune cells, thereby altering immune cell signaling thresholds [41]. These findings suggest that specific lipid subtypes such as LP(a), rather than general lipid elevations, may have unique relevance in the immunopathogenesis of MG.

Beyond disease mechanisms, the role of MG treatments in modulating lipid profiles is clinically relevant. As previously noted, Zhang et al. (2024) identified DL as an adverse event in 3.6% of steroid-resistant MG patients treated with Tacrolimus [41]. Tacrolimus, a calcineurin inhibitor commonly used in refractory autoimmune diseases and transplant medicine, is associated with well-documented metabolic side effects. These include hyperlipidemia, insulin resistance, and new-onset diabetes mellitus, believed to result from impaired pancreatic β-cell function, mitochondrial dysfunction, and suppression of insulin gene transcription. Furthermore, calcineurin inhibition has been shown to disrupt hepatic lipid metabolism and impair cholesterol efflux mechanisms, leading to elevated circulating levels of LDL cholesterol and triglycerides [41]. In contrast, Li et al. (2018) in a cohort of 102 glucocorticoid-naïve MG patients (PwMG) with a mean age of 40 years reported a high prevalence of both glucose and metabolic derangements [20]. Specifically, DL was present in 50% of male PwMG and 25% of female PwMG, a finding that may be attributed to insulin resistance secondary to muscle weakness. This raises the possibility that intrinsic disease mechanisms, independent of iatrogenic steroid exposure, may predispose certain PwMG to metabolic disturbances. One plausible explanation is the interplay between chronic muscle weakness and reduced physical activity, which in turn may promote insulin resistance and dysregulated lipid metabolism.

Of additional interest is the phenotypic variability in DL prevalence across MG subtypes. Di Stefano et al. (2024) reported a higher prevalence of DL in patients with ocular MG compared to those with generalized MG (gMG) [32]. This finding may reflect variations in treatment exposure, physical activity, or autonomic involvement, as patients with ocular MG often retain greater overall mobility, which could differentially influence their metabolic profile compared to those with generalized MG.

Another clinically relevant aspect is the effect of lipid-lowering therapies, particularly statins, on MG disease activity. While statins are widely prescribed for the primary and secondary prevention of cardiovascular disease, they have been increasingly implicated in the exacerbation of MG symptoms in susceptible individuals. Case reports and observational studies have described new-onset MG or worsening of preexisting symptoms shortly after statin initiation. One proposed mechanism is the “double-hit phenomenon”, wherein the antibody-mediated post-synaptic acetylcholine receptor dysfunction characteristic of MG is compounded by statin-induced myopathy, leading to additive neuromuscular impairment [42]. At the molecular level, statins inhibit HMG-CoA reductase, a key rate-limiting enzyme in the mevalonate pathway responsible for cholesterol and isoprenoid biosynthesis. This inhibition may reduce the availability of essential intermediates such as farnesyl pyrophosphate and geranylgeranyl pyrophosphate, resulting in impaired mitochondrial function, muscle cell apoptosis, and reduced levels of prenylated proteins critical for myocyte integrity [43]. Additionally, emerging evidence suggests that statins modulate immune function by altering T-cell polarization—shifting the immune response toward a T-helper 2 (TH2) phenotype at the expense of T-helper 1 (TH1) cells. While this TH2 predominance is generally considered anti-inflammatory, in the context of MG it may paradoxically enhance B-cell activation and antibody production, thus exacerbating autoimmune activity [16,43].

Although the growing recognition of the interplay between metabolic and autoimmune conditions is promising, substantial gaps persist in the current literature regarding the exact prevalence, risk factors, and consequences of DL in MG. Our study addresses this gap by providing, to our knowledge, the first systematic review and meta-analysis estimating DL prevalence in the MG population. This work emphasizes the importance of a multidisciplinary approach in managing PwMG, integrating neurological, metabolic, and cardiovascular care. By estimating the prevalence of DL in the MG population, our study not only enriches current research on metabolic comorbidities associated with MG but also offers valuable insights into potential shared pathophysiological mechanisms, disease burden, and the necessity for integrated management strategies to optimize long-term outcomes. Further stratification by disease subtype, sex, age, and APOE genotype could yield greater analytical precision and reveal subtype-specific associations. Ultimately, understanding the bidirectional relationship between lipid dysregulation and MG pathogenesis may unlock new therapeutic targets and preventive strategies, enhancing the quality of life and functional outcomes for this unique patient population.

### Limitations

This study has certain limitations to be acknowledged. To begin with, there is a high degree of heterogeneity among the included studies. Moreover, given that the majority of included studies reported DL as a demographic characteristic, there was no stratification based on MG type, MG classification, or type of treatments. Third, there was a lack of data regarding the type of diagnostic criteria used for the confirmation of DL. Lastly, given the prevalence of DL in the GP and the absence of case–control and longitudinal studies, current evidence does not allow for the establishment of causality or directionality in the relationship between MG and DL. Despite the aforementioned limitations, our findings serve as a crucial first step, laying the groundwork for future research to refine our understanding of the MG-DL relationship and optimize management strategies.

## 5. Conclusions

The results of this study highlight the prevalence and potential pathophysiological interplay between DL and MG. Disturbed lipid metabolism and dyslipidemia (DL) have been observed in several autoimmune conditions, such as systemic lupus erythematosus—primarily associated with inflammation and organ involvement—and rheumatoid arthritis (RA), where chronic inflammation alters the composition and structure of HDL particles. These changes diminish HDL’s anti-atherogenic properties and promote LDL oxidation and atherosclerotic plaque formation [44,45]. In our pooled cohort, approximately one in four PwMG had comorbid DL, with the age of MG onset emerging as a key factor influencing DL prevalence. Based on the current literature, a critical concern in managing DL in PwMG is the use of statins, which have been shown to exacerbate MG symptoms and increase the risk of myasthenic crisis, underlying the need for appropriate therapeutic strategies. In this context, PCSK9 inhibitors present a promising treatment option for DL in MG patients, offering a safer lipid-lowering approach without the adverse effects associated with statins. Additionally, the potential shared mechanisms linking MG and DL, such as the role of LP(a), suggest that routine monitoring of LP(a) levels in MG patients could enable earlier detection and allow for more personalized interventions. These findings emphasize the significance of personalized intervention strategies for DL in PwMG, incorporating alternative lipid-lowering therapies and leveraging routine biomarkers like LP(a) to facilitate early detection and individualized treatment plans, ultimately optimizing clinical outcomes. Finally, structured, large-scale case–control and longitudinal studies are needed in order to examine the directionality and causality between MG and DL.

## Figures and Tables

**Table 1:** Included studies.

Author	Country	Sample	Events	Age	Disease Duration	Onset Age
Oh et al., 2008 [16]	USA	170	54	58.7	10	48.7
Haggård et al., 2013 [17]	Sweden	59	10	-	10	-
Liu et al., 2017 [18]	China	2195	71	41.1	-	-
Machado-Alba et al., 2017 [19]	South America	306	41	53	-	-
Li et al., 2018 [20]	China	102	38	40	-	-
Tanovska et al., 2018 [21]	Rep. of Macedonia	127	14	-	-	48.8
Misra et al., 2019 [22]	India	81	27	42	-	-
Chu et al., 2019 [34]	Taiwan	349	91	44	10	-
Aleksić et al., 2021 [23]	Serbia	687	189	55	-	-
Johnson et al., 2021 [24]	USA	39	4	60	-	-
Mahic et al., 2022 [25]	UK	456	99	54.5	34.1	50.5
Philips et al., 2022 [26]	USA	41,940	12,415	64.7	-	-
Zhou et al., 2022 [27]	China	69	5	-	2.8	44.7
Qi et al., 2022 [28]	USA	38,399	14,948	63.5	-	-
Digala et al., 2022 [29]	USA	91	41	64.3	-	-
Ozdemir et al., 2023 [30]	Turkey	168	27	55.9	-	45.8
Tsai et al., 2024 [31]	Taiwan	2813	699	-	-	-
Di Stefano et al., 2024 [32]	Italy	178	23	59.2	-	-
Qi et al., 2025 [33]	USA	10,718	7024	-	-	70.4

## Data Availability

All data are available in plain text format upon reasonable request.

## Supplementary Materials

The following supporting information can be downloaded at: https://www.mdpi.com/article/10.3390/medicina61061067/s1, File S1: Search algorithms; Figure S1: funnel plot; Figure S2: Risk of bias assessment.
