# Supplementary material for: Dyslipidemia in Myasthenia Gravis: A Systematic Review and Meta-Analysis
Source: Medicina (Kaunas). 2025 Jun 10;61(6):1067. doi: 10.3390/medicina61061067 (PMC12195350; doi:10.3390/medicina61061067)

Dyslipidemia in Myasthenia Gravis:

A systematic review and meta-analysis.

Supplementary-Materials

Search Algorithms

- MedLine PubMed

(myasthenia gravis) AND ((dyslipidemia) OR (hyperlipidemia) OR (cardiovascular) OR (metabolic syndrome))

- Scopus

“Myasthenia gravis” AND (“dyslipidemia” OR “hyperlipidemia” OR “cardiovascular” OR “metabolic syndrome”)

Supplementary Figure Legends

figure S1: Robins-E traffic light plot

figure S2: Funnel plot for publication bias

figure S1: Robins-E traffic light plot

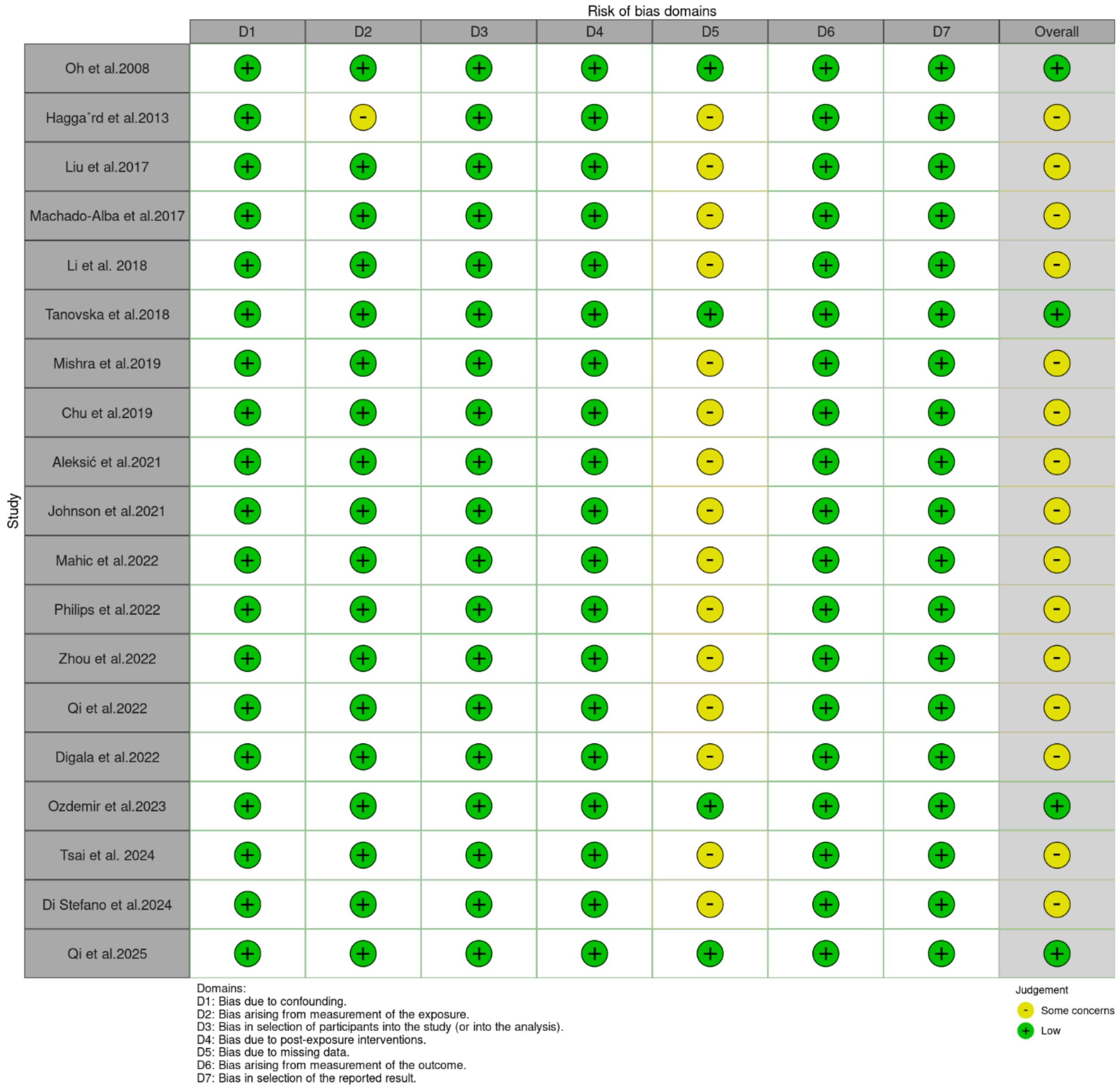

figure S2: Funnel Plot for publication bias

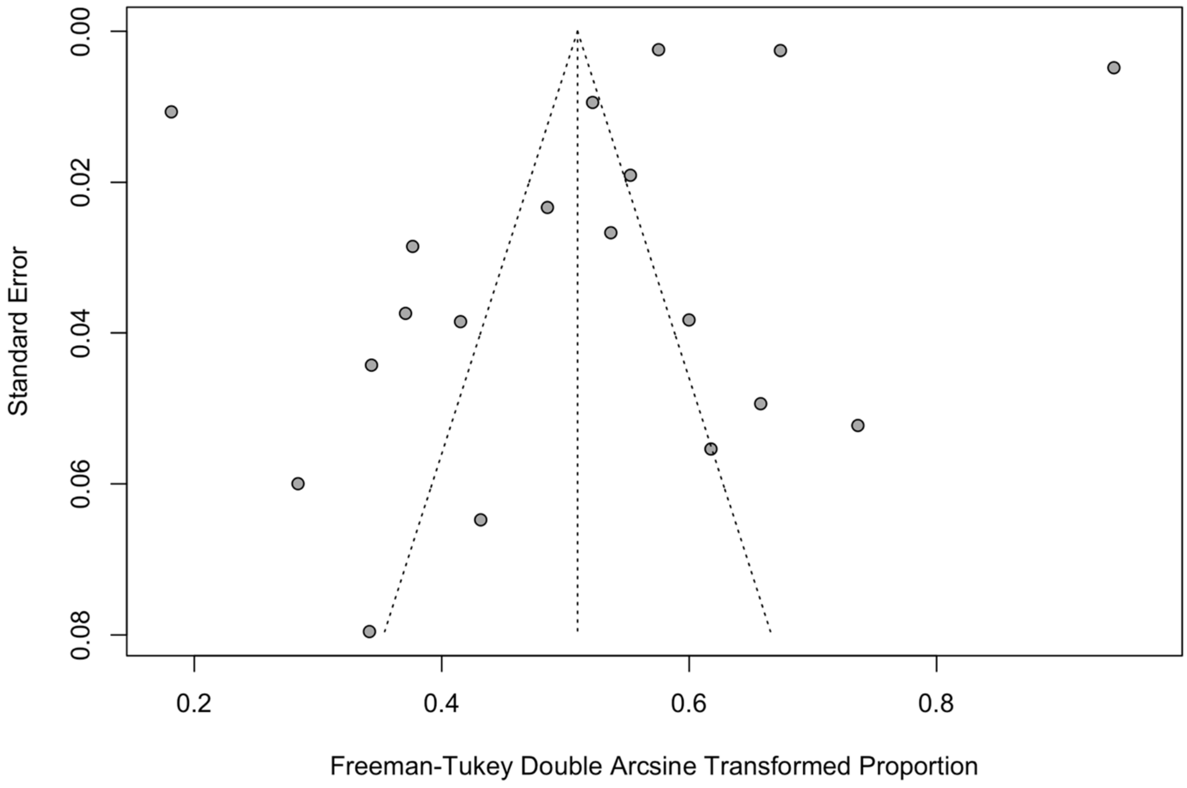

Supplement: Supplementary file 1 [file medicina-61-01067-s001.zip › medicina-3651071-supplementary.pdf]
